# Novel Technique of Interproximal Enamel Reduction Based on Computer-Aided Navigation Technique—An In Vitro Study

**DOI:** 10.3390/jpm14020138

**Published:** 2024-01-26

**Authors:** María Dolores Cotrina-Peregrín, Patricia Arrieta-Blanco, Juan Manuel Aragoneses-Lamas, Alberto Albaladejo Martínez, Ana Belén Lobo Galindo, Álvaro Zubizarreta-Macho

**Affiliations:** 1 Doctoral Student in Cancer Biology and Clinic and Translational Medicine program, Faculty of Medicine, University of Salamanca, 37008 Salamanca, Spain; mcotrper@uax.es; 2 Faculty of Health Sciences, Alfonso X el Sabio University, 28691 Madrid, Spain; parribla@uax.es (P.A.-B.); jaraglam@uax.es (J.M.A.-L.); 3 Department of Dentistry, Universidad Federico Henríquez y Carvajal, Santo Domingo 10106, Dominican Republic; 4 Department of Surgery, Faculty of Medicine, University of Salamanca, 37008 Salamanca, Spain; albertoalbaladejo@usal.es (A.A.M.); alobogal@usal.es (A.B.L.G.)

**Keywords:** orthodontics, interproximal enamel reduction, standard tessellation language, digital impression, computer-aided static system

## Abstract

The aim of this study was to analyze and compare the accuracy of a novel interproximal enamel reduction (IPR) technique based on a computer-aided static navigation technique with respect to a conventional free-hand-based technique for interproximal enamel reduction. Twenty anatomical-based experimental cast models of polyurethane were randomly distributed into the following IPR techniques: IPR technique based on computer-aided static navigation technique (*n* = 10) (GI) for Group A and conventional free-hand-based technique for the IPR (*n* = 10) (FHT) for Group B. The anatomical-based experimental cast models of polyurethane randomly assigned to the GI study group were submitted for a preoperative 3D intraoral surface scan; then, datasets were uploaded into 3D implant-planning software to design virtual templates for the interproximal enamel reduction technique. Afterward, the anatomical-based experimental cast models of polyurethane of both GI and FHT study groups were subjected to a postoperative digital impression by a 3D intraoral surface scan to compare the accuracy of the interproximal enamel reduction techniques at the buccal (mm), lingual/palatal (mm), and angular (◦) levels using the Student *t*-test. Statistically significant differences between the interproximal enamel reduction technique based on the computer-aided static navigation technique and the conventional free-hand-based technique for the interproximal enamel reduction at the buccal (*p* = 0.0008) and lingual/palatal (*p* < 0.0001) levels; however, no statistically significant differences were shown at the angular level (*p* = 0.1042). The interproximal enamel reduction technique based on computer-aided static navigation technique was more accurate than the conventional free-hand-based technique for interproximal enamel reduction.

## 1. Introduction

Interproximal enamel reduction (IPR) or “stripping” is an irreversible technique used to reduce controlled amounts of enamel on the proximal surface of the tooth by decreasing its mesiodistal size as an alternative to extraction or expansion in borderline cases [1]. This procedure is recommended to increase the space in the dental arch and align the teeth [2] without an excessive labioversion of incisors or, in some cases, without increasing the intercanine distance, solving many malocclusions caused by dental crowding generated by the difference between the size of the teeth and the length of the dental arch [3]. The IPR is indicated in cases of not very severe crowding, from 4 to 8 mm, to align teeth without retruding the profile and reduce the unsightly interproximal black triangles in gingival improving the periodontal prognosis [3,4,5]. The thickness of the enamel is greater in the posterior than in the anterior region and a little greater in the distal face than in the mesial (0.10 mm (95% CI, 0.09–0.12)), and there is even more in molars than in premolars (0.12 mm (95% CI, 0.07–0.17)). In addition, it is important to highlight that in temporary teeth, the enamel layer is less than in permanent teeth, and they also have a greater tendency to demineralize than permanent teeth due to the low mineral content and high organic content [6].

Stripping procedures can be performed through manual stainless-steel strips, diamond blades, motor-driven abrasive strips (strips connected to a micromotor), or air rotor stripping (milling cutter connected to a turbine). Sheridan and Chudasama created action guides with this last painless, fast, and efficient technique, being able to perform an interproximal reduction of 1 mm at each point of contact in the posterior area and no more than 0.5 mm in the anterior area because the enamel is thinner. After using bur, it is recommended to finish with thin rotating discs Sof-Lex to soften and contour the tooth in the interproximal area. Therefore, these authors also use a thin abrasive strip impregnated with 35% orthophosphoric acid and a water syringe. Finally, they recommend applying fluoride because the reduced enamel surface tends to re-mineralize and when applying fluorinated products, they prevent caries in this area [5]. It is also very important to use cooling water and suction even if visibility decreases to prevent the temperature from rising [7,8,9]. Moreover, Gazzani et al. analyzed the effects on enamel surface after reducing the interproximal enamel surface by the oscillating mechanical system and reported more regular enamel surfaces using a single metallic strip and recommended adequate polishing after IPR procedures to maintain the enamel morphologic integrity [10]. Additionally, Kaauara et al. evaluated the enamel surface after IPR and recommended minimizing the number of abrasions caused by stripping to obtain a surface condition close to an untreated enamel surface using Soft-Lex abrasive discs to obtain a shiny finish and polish of the entire interdental surface [11].

Surgical guides are indispensable biomedical devices for the proper insertion of dental implants. In particular, Sarment et al. highlighted the effectiveness of CAD/CAM stereolithographic rapid prototyping techniques for the manufacturing of surgical guides to increase the accuracy of dental implant placement [10]. In general, diagnostic and surgical guides must have properties of rigidity, stability, and precision to ensure accuracy and safety during implant insertion procedures [11]. Additionally, CAD-CAM procedures require high cost and a detailed and precise planning process, and treatment plan. As a result, it allows the manufacture of personalized guidance templates for minimally invasive treatment and allows accurate rehabilitation. Moreover, Gao JH (2021) assessed the accuracy between different tooth preparation guides for veneer restorations and concluded that personalized guidance templates improved the accuracy of tooth preparation [12].

Furthermore, previous measurement techniques have been used to quantify the amount of enamel reduced such as the optical microscope, the transmission electron microscope (TEM) [13], the scanning electron microscope (SEM) [14], the atomic force microscope (AFM) [15], the standardized intraoral electron photography technique, the electronic photograph measurement technique, and the intraoral scanners [16,17]. Moreover, the roughness of the enamel has been analyzed by prolymphometry [18], although it has been also measured with the scanning electron microscope (SEM) and the atomic force microscope (AFM) [19].

The aim of this study was to analyze and compare the accuracy of a novel interproximal enamel reduction technique based on the computer-aided static navigation technique with respect to a conventional free-hand-based technique for the interproximal enamel reduction, with a null hypothesis (H_0_) stating that there are no differences between the accuracy of a novel interproximal enamel reduction technique based on computer-aided static navigation technique and the conventional free-hand-based technique for the interproximal enamel reduction.

## 2. Material and Methods

### 2.1. Study Design

A randomized controlled experimental trial was conducted in accordance with the principles defined in the International Organization for Standardization (ISO 14801) [20] at the Department of Surgery of the University of Salamanca (Salamanca, Spain), the Stomatology Department of Fundación Jimenez Díaz Hospital (Madrid, Spain) and the Department of Biomedical, Surgical and Dental Sciences of Universitá degli Studi di Milano, (Milan, Italy), between September 2022 and October 2023. In addition, this study was authorized by the Ethical Committee of the Faculty of Health Sciences, University Alfonso X el Sabio (Madrid, Spain) in July 2022 (Process No. 14/2022). Two hundred (200) interproximal enamel reduction procedures were included in this study to ensure a power effect of 80.00% for detecting statistically significant differences. The bilateral Student’s t-test of two independent samples was used to evaluate the null hypothesis H₀: μ₁ = μ₂, with a significance level of 5.00%. The sample size calculation was carried out on the lingual/palatal level variable; to detect differences of 0.2 units with a deviation of 0.1, 10 observations per group were needed.

### 2.2. Experimental Procedure

Twenty anatomical-based experimental cast models of polyurethane (Ref. 1522-62; Sawbones Europe AB; Malmo, Sweden), with contact points between adjacent teeth, were used in this study. Afterward, the anatomical-based experimental models of polyurethane were randomly distributed (Epidat 4.1, Galicia, Spain) into the following measurement techniques: interproximal enamel reduction technique based on computer-aided static navigation technique (NemoStudio^®^, Nemotec, Madrid, Spain) (n = 10) (guided IPR (GI)) for Group A and conventional free-hand-based technique for the interproximal enamel reduction (n = 10) (Free-hand IPR (FHT)) for Group B. The use of polyurethane was based on the American Society for Testing and Materials’ (ASTM F-1839-08) approval of the use of polyurethane for testing instruments and dental procedures (“Standard Specification for Rigid Polyurethane Foam for Use as a Standard Material for Test Orthopedic Devices for Instruments”) [21].

### 2.3. Interproximal Enamel Reduction Procedure

Afterward, the anatomical-based upper and lower experimental cast models of polyurethane were fixed in a phantom imitating the patient’s head and subsequently attached to a dental chair to simulate a real setting. Then, an interproximal enamel reduction of 0.2 mm width was performed using air-rotor burs with 7 mm head length, a total length of 23.5 mm, and a minimum and maximum diameter of 0.2 mm and 0.55 mm, respectively (Code 852-005, E11S Komet Medical, Lemgo, Germany), fixed to the high-speed rotation device (Tornado LK; Bien Air, Le Noirmont, Switzerland) placed on the dental chair at 410,000 rpm with profuse irrigation. In particular, one bur was used on each interproximal enamel reduction technique.

**Figure 1:**
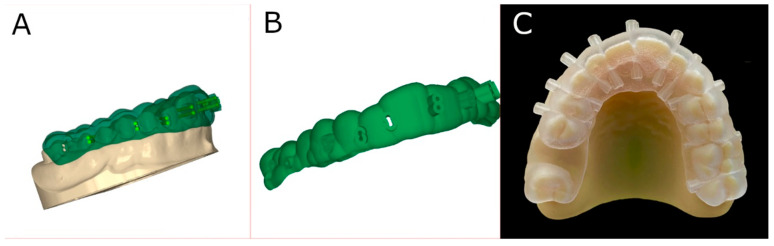
(**A**) Virtual implant drills designing crossing the contact point at the interproximal surface of the teeth, (**B**) virtual template designing, and (**C**) stereolithographic template attached on the GI experimental cast models of polyurethane.

The anatomical-based experimental cast models of polyurethane randomly assigned to the GI study group were submitted for a 3D intraoral surface scan (True Definition, 3M ESPE™, Saint Paul, MN, USA) for a digital impression. Datasets obtained from the digital workflow were uploaded into 3D implant-planning software (NemoStudio^®^, Nemotec, Madrid, Spain) to design virtual templates for interproximal enamel reduction technique based on computer-aided static navigation technique. Then, two virtual implant drills were designed by crossing the contact point at the interproximal surface of the teeth with a diameter and length of 0.2 and 23.5 mm, respectively, according to the air-rotor bur measurements (Figure 1A) from the recommendations established by Chudasama et Sheridan (2007) [22]. In particular, the bur was placed perpendicular to the axial shaft of the adjacent teeth by buccal to lingual movements up to remove 0.2 mm on mesial and 0.2 mm on distal surface of the adjacent teeth under profuse irrigation. Ten interproximal enamel reduction procedures were performed in each anatomical-based experimental cast model of polyurethane. After designing the virtual templates (Figure 1A,B), they were fabricated using the stereolithography technique (ProJet 6000, 3D Systems, Rock Hill, SC, USA) (Figure 1C). The templates fit the model and did not need further adjustments.

The anatomical-based experimental cast models of polyurethane randomly assigned to the FHT study group were subjected to interproximal enamel reduction procedures following the recommendations established by Chudasama et Sheridan [5] by placing the bur perpendicular to the axial shaft of the adjacent teeth by buccal to lingual movements up to remove 0.2 mm on mesial and 0.2 mm on distal surface of the adjacent teeth under profuse irrigation.

The interproximal enamel reduction procedures were performed by a unique operator with more than 20 years of experience in orthodontics.

### 2.4. Digital Measurement Procedure

**Figure 2:**
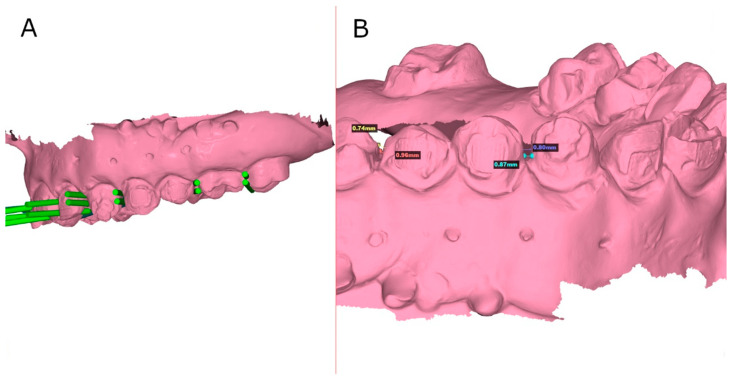
(**A**) Alignment procedure between preoperative planning (green cylinders) and postoperative STL digital files. (**B**) Lineal measurements of the interproximal enamel reduction procedures.

Afterward, the anatomical-based experimental cast models of polyurethane of both GI and FHT study groups were submitted to a postoperative digital impression by an intraoral scan (True Definition, 3M ESPE™, Saint Paul, MN, USA) via a 3D in-motion video imaging technology to generate an STL digital file using a cloud of points that create a tessella network, representing three-dimensional objects as polygons composed of equilateral triangle tessellas [5,23]. The capturing images procedure was performed following manufacturer recommendations by scanning the incisal/occlusal plane and the vestibular and lingual surfaces. Afterward, the preoperative and postoperative “Standard Tesellation Language” (STL) digital files were imported to a 3D implant-planning software (NemoStudio^®^, Nemotec, Madrid, Spain); a full-arch alignment procedure was conducted. The preoperative STL digital file was considered the reference digital file, and the postoperative STL digital file was superimposed on it using the buccal and palatal/lingual surfaces of the anterior teeth and the occlusal, buccal, and palatal/lingual surfaces of the posterior teeth with the best-fit algorithm (Figure 2A). Afterward, the accuracy of the interproximal enamel reduction was measured at buccal (mm), lingual/palatal (mm), and angular level (◦). This digital measurement procedure was performed in a previous study of Triduo et al. [24]. Additionally, interproximal enamel reduction distance was also measured (Figure 2B).

### 2.5. Statistical Tests

Statistical analysis of the measurement variables was conducted using SAS 9.4 (SAS Institute Inc., Cary, NC, USA). Descriptive statistics are expressed as mean and SD for the interproximal enamel reduction (mm). Comparative analysis between the interproximal enamel reduction technique based on the computer-aided static navigation technique and the conventional free-hand-based technique for the interproximal enamel reduction at buccal (mm), lingual/palatal (mm), and angular (◦) levels was analyzed by using the Student *t*-test and the Mann–Whitney non-parametric test. The repeatability and reproducibility of the digital measurement technique were analyzed using Gage R&R statistical analysis.

## 3. Results

**Figure 3:**
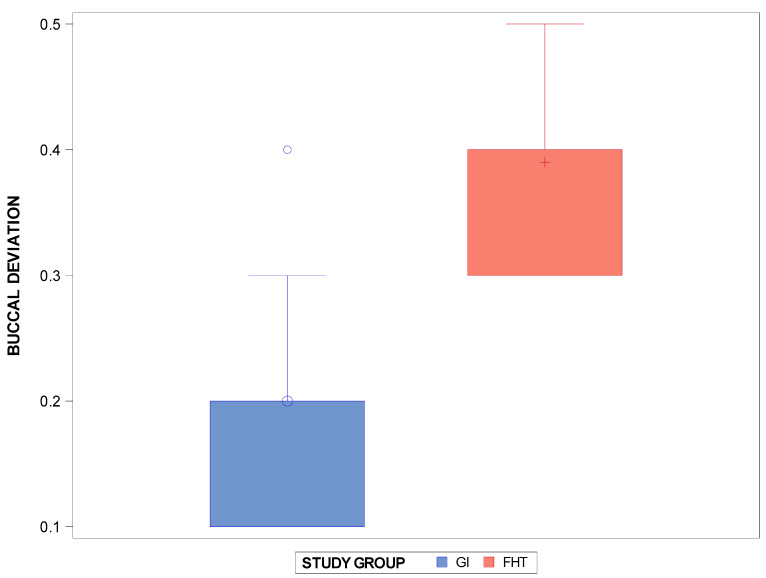
Box plots of the buccal deviation of the interproximal enamel reduction (mm) between the computer-aided static navigation technique and the conventional free-hand-based technique. The horizontal line in each box represents the median value. +,◦: represent the mean value.

The means and SD values for the interproximal enamel reduction (mm) between the interproximal enamel reduction technique based on the computer-aided static navigation technique and the conventional free-hand-based technique for the interproximal enamel reduction at the buccal level are displayed in Table 1 and Figure 3.

The Student *t*-test showed statistically significant differences (*p* = 0.0008) between the interproximal enamel reduction technique based on the computer-aided static navigation technique (0.20 ± 0.09 mm) and the conventional free-hand-based technique (0.39 ± 0.07 mm) for the interproximal enamel reduction at the buccal level (Figure 3).

**Figure 4:**
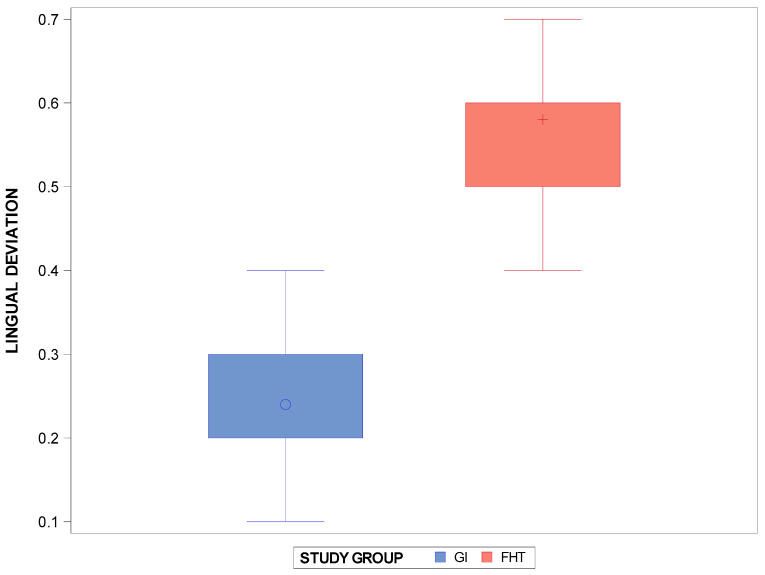
Box plots of the lingual/palatal deviation in the interproximal enamel reduction (mm) between the computer-aided static navigation technique and the conventional free-hand-based technique. The horizontal line in each box represents median value. +,◦: represent the mean value.

The Student *t*-test showed statistically significant differences (*p* < 0.0001) between the interproximal enamel reduction technique based on the computer-aided static navigation technique (0.24 ± 0.11 mm) and the conventional free-hand-based technique (0.58 ± 0.9 mm) for the interproximal enamel reduction at the lingual/palatal level (Figure 4).

**Figure 5:**
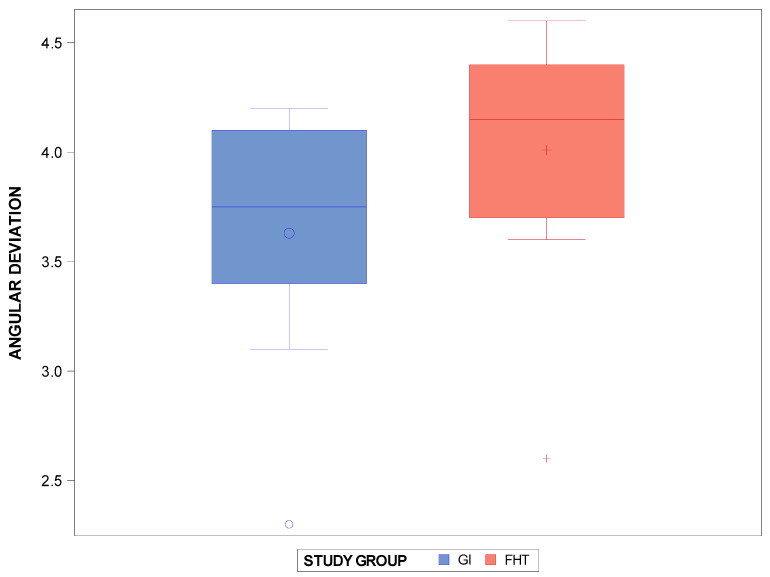
Box plots of the angular deviation of the interproximal enamel reduction (°) between the computer-aided static navigation technique and the conventional free-hand-based technique. The horizontal line in each box represents median value. +,◦: represent the mean value.

The Student *t*-test showed statistically significant differences (*p* = 0.01042) between the interproximal enamel reduction technique based on the computer-aided static navigation technique (3.36 ± 0.58°) and the conventional free-hand-based technique (4.01 ± 0.60°) for the interproximal enamel reduction at the angular level (Figure 5).

## 4. Discussion

The results presented in this study rejected the null hypothesis (H_0_) that states there are no differences between the accuracy of a novel interproximal enamel reduction technique based on a computer-aided static navigation technique and a conventional free-hand-based technique for the interproximal enamel reduction.

Sittikornpaiboon et al. assessed the accuracy of computer-assisted implant surgery through surgical templates based on the CBCT scan and STL digital files obtained by digital impressions, concluding that this digital workflow is sensitive to the milling protocol and the design of the device [25]. In addition, it has been reported that fully guided implant surgery is more accurate than partially guided implant surgery and that deviation of the dental implant position may be influenced by the dental implant location; however, it is not affected by implant systems, dental implant software [26], or the manufacturing process of the surgical templates by fused deposition modeling printed in-office or by stereolithography [27]. Moreover, tooth-supported computer-aided static implant surgery by surgical templates has evidenced a predictable treatment outcome for dental implant placement, showing statistically significant differences (*p* < 0.05) with respect to the number of teeth [28]. Moreover, fixation pins have been also recommended to attach the surgical templates to the maxilla during the drilling procedure; however, Pessoa et al. (2022) reported that the use of surgical templates with or without fixing pins for dental implant placement provided predictable treatment outcomes [29]. In the present study, the surgical template designed for the interproximal enamel reduction procedure did not include fixing pins because it was tooth-supported by fully dentated anatomical-based upper and lower experimental cast models; additionally, the operator checked the surgical template stability before use. In resume, Ngamprasertkit et al. (2022) highlighted that computer-aided implant surgery through fully digital workflow is a practical procedure that provides an accurate dental implant placement [30].

Afterward, the promising results associated with the computer-aided surgical implant technique led to its application to tooth preparation procedures. In particular, Li et al. (2020) reported that 3D-printed surgical templates improved the control and management of the reduction depth of veneer preparations, increasing the accuracy compared to guide milling cutters [31]. Additionally, Jurado et al.’s (2021) custom-fenestrated metal guides have also been used to selectively reduce tooth surface [32]. Moreover, Zong Yi et al. (2020) used a 3D-printed metal alloy guide, which allowed greater accuracy than measuring cutters [33]. Furthermore, Gao JH (2021) analyzed the degree of accuracy between different preparation guides for veneer restorations and highlighted the relevance of using tooth-preparing guides to achieve accurate tooth preparation (Group F was significantly higher than the rest) [34]. In addition, Johner et al. performed an in vitro study to evaluate the predictability of the expected amount of IPR using three common stripping devices on premolar teeth and concluded that for all scenarios, the amount of stripping was less than the predetermined and expected. However, the authors highlighted that traditional hand-held abrasive strips performed an unpredictable IPR in posterior teeth, and motor-driven devices reduce more enamel at the contact point, so this will be flatter and might even show a little edge around the stripped area. In this case, it will be necessary to smooth the edges and reshape the contact point with further devices such as diamond burs [33].

In recent years, numerous studies have been carried out to investigate the amount of enamel tissue removed during stripping procedures, compare the reduction in the distal versus mesial surface, and compare the amount of stripping performed with respect to the planned different digital measurement methods, including the Invisalign Clincheck software [33], the digital set up in the treatment with clear aligner devices [14], and Orthocad digital software [35]. In addition, Kalemaj and Levrini compared the programmed and implemented interproximal enamel reduction in a clinical setting and reported a statistically significant mean difference of 0.15 mm (SD ± 0.14 mm; *p* = 0.0001). These authors measured the differences in the Orthocad digital software; however, the IPR procedures were performed by free-hand technique without computer-aided static navigation techniques [36]. These authors did not analyze the reliability of the measurement technique and quantified the mesiodistal distances of each tooth, from second premolar to second premolar. However, posterior teeth have been recommended as the enamel thickness increases in these teeth despite presenting worse accessibility [37]

The present study selected the True Definition intraoral since Guth et al. reported that True Definition showed higher trueness (21.8 µm) than Cerec Bluecam (34.2 µm), Cerec Omnicam (43.3 µm), Itero (49.0 µm), Lava C.O.S. (47.7 µm), TRIOS (25.7 µm), and TRIOS color (26.1 µm) digital impression systems for dental nature arch scanning [38]. Furthermore, the present study includes some limitations since Jivanescu et al. reported that the presence of adjacent teeth can decrease the view of interproximal surfaces [36]. Additionally, the full-arch scanning may introduce higher deviations than partial-arch scanning [39], ambient temperature [40], number of teeth and location [41], scanning time [42], lighting conditions [43], and humidity [44]; therefore, further clinical studies are encouraged to increase the results of these procedures.

Meredith et al. analyzed the enamel nanotopography after interproximal enamel reduction using atomic force microscopy (AFM) and reported that the enamel surface becomes progressively smoother from burs to strips and discs to polishers. In addition, surface roughness was higher using a medium roughness strip bur (707 nm) and decreased using medium strip bands (501 nm), fine strip burs (407 nm), fine strip bands (318 nm), mesh strip discs (307 nm), curved strip discs (224 nm), and a Sof-Lex polishing device (37 nm) [39]. However, these measurement procedures did not provide information related to the accuracy of the interproximal enamel reduction procedures or the hard tissue (enamel and/or dentin) affected by tooth preparation. These measurement procedures provide 2D information on a selected area and do not provide 3D information related to the profiles and geometry of the tooth after the interproximal enamel reduction technique.

## 5. Conclusions

The results show that the interproximal enamel reduction technique based on computer-aided static navigation technique was more accurate than the conventional free-hand-based technique for interproximal enamel reduction.

## Figures and Tables

**Table 1:** Descriptive statistics of the interproximal enamel reduction (mm) between the interproximal enamel reduction technique based on computer-aided static navigation technique and the conventional free-hand-based technique for the interproximal enamel reduction at buccal level.

Measure	Technique	*n*	Mean (mm)	SD (mm)	Minimum (mm)	Maximum (mm)
Buccal	GI	100	0.20	0.09	0.10	0.40
	FHT	100	0.39	0.07	0.30	0.50
Lingual/Palatal	GI	100	0.24	0.11	0.10	0.40
	FHT	100	0.58	0.9	0.40	0.70
Angular	GI	100	3.63	0.58	2.30	4.20
	FHT	100	4.01	0.60	2.60	4.60

SD: standard deviation.

## Data Availability

Data are available upon request due to restrictions, e.g., privacy or ethical reasons.
